# Multiple Microinvasion Foci in Ductal Carcinoma *In Situ* Is Associated With an Increased Risk of Recurrence and Worse Survival Outcome

**DOI:** 10.3389/fonc.2020.607502

**Published:** 2020-12-03

**Authors:** Jing Si, Rong Guo, Huan Pan, Xiang Lu, Zhiqin Guo, Chao Han, Li Xue, Dan Xing, Wanxin Wu, Caiping Chen

**Affiliations:** ^1^ Department of Breast Disease, The First Hospital of Jiaxing & The Affiliated Hospital of Jiaxing University, Jiaxing, China; ^2^ Cancer Research Center, The First Hospital of Jiaxing & The Affiliated Hospital of Jiaxing University, Jiaxing, China; ^3^ Department of Breast Surgery, Breast Cancer Center of the Third Affiliated Hospital of Kunming Medical University, Cancer Hospital of Yunnan Province, Kunming, China; ^4^ Department of Central Laboratory, The First Hospital of Jiaxing & The Affiliated Hospital of Jiaxing University, Jiaxing, China; ^5^ Department of Pathology, The First Hospital of Jiaxing & The Affiliated Hospital of Jiaxing University, Jiaxing, China

**Keywords:** ductal carcinoma *in situ*, microinvasion, recurrence, foci, survival outcome

## Abstract

**Background:**

Ductal carcinoma *in situ* with microinvasion (DCISM) was defined as one or more foci of invasion beyond the basement membrane within 1 mm. The size of primary lesion is associated with axillary status and prognosis in patients with invasive breast cancer; thus, it is of interest to determine whether multiple foci of microinvasion are associated with a higher risk of positive axillary status or worse long-term outcomes in patients with DCISM.

**Methods:**

This study identified 359 patients with DCISM who had undergone axillary evaluation at our institute from January 2006 to December 2015. Patients were categorized as one focus or multiple foci (≥2 foci) according to the pathological results. Clinicopathological features, axillary status, and disease-free survival rate were obtained and analyzed.

**Results:**

Of 359 patients, 233 (64.90%) had one focus of microinvasion and 126 (35.10%) had multiple foci. Overall, 242 (67.41%) and 117 (32.59%) patients underwent sentinel lymph nodes biopsy (SLNB) and axillary lymph nodes dissection (ALND), respectively. Isolated tumor cells were found in four (1.11%) patients and axillary metastasis rate was 2.51%. Neither axillary evaluation methods (*P* = 0.244) nor axillary metastasis rate (*P* = 0.559) was significantly different between patients with one focus and multiple foci. In univariate analysis, patients with multiple foci tended to have larger tumor size (*P* < 0.001), higher nuclear grade (*P* = 0.001), and higher rate of lymphatic vascular invasion (*P* = 0.034). Also, the proportion of positive HER2 (*P* = 0.027) and Ki67 level (*P* = 0.004) increased in patients with multiple foci, while in multivariate analysis, only tumor size showed significant difference (*P* = 0.009). Patients with multiple foci were more likely to receive chemotherapy (56.35 *vs* 40.77%; *P* = 0.028). At median 5.11 years follow-up, overall survival rate was 99.36%. Patients with multiple microinvasive foci had worse disease-free survival rate compared with one-focus patients (98.29 *vs* 93.01%, *P* = 0.032).

**Conclusion:**

Even though the numbers of microinvasion were different and patients with multiple foci of microinvasion tended to have larger tumor size, there was no higher risk of axillary involvement compared with patients with one focus of microinvasion, while patients with multiple microinvasive foci had worse DFS rate. Thus, DCISM patients with multiple foci of microinvasion may be the criterion for more aggressive local–regional treatment. Optimization of adjuvant therapy in DCISM patients is required.

## Introduction

Owing to the widespread adoption of screening for breast cancer and the improvements in the sensitivity of mammography, the diagnosis of ductal carcinoma *in situ* (DCIS) has increased dramatically over the past few decades according to the statistics (1). Due to the increase proportion of DCIS, a rare diagnosis called DCIS with microinvasion (DCISM) is also increasing. According to the staging system of the American Joint Committee on Cancer (AJCC), DCISM was defined as DCIS with one or more microscopic foci of invasion beyond the basement membrane within 1 mm in the longest diameter, which is identified in 10–20% of DCIS cases and approximately 1% of all breast cancers (2, 3). Patients with DCISM are generally considered to have a better prognosis than IDC, while worse than pure DCIS (4, 5).

The rarity of DCISM has made studies difficult, and the few studies of axillary management and outcomes in DCISM showed controversial results (6–14). Previous studies showed that the size of primary lesion is associated with axillary positivity and prognosis in patients with invasive breast cancer (15). Moreover, small, single institute studies have showed that the extent of microinvasion may be a predictive risk for axillary metastasis and worse prognosis (16). Thus, it is of interest to determine whether multiple foci of microinvasion is associated with a higher risk of positive axillary status or worse long-term outcomes in patients with DCISM.

We hypothesized that in the cohort of patients with DCISM, a significant association of multiple foci of microinvasion with axillary metastasis and worse prognosis might exist. In the current study, we report the incidence of axillary involvement and disease-free survival (DFS) in DCISM patients with a single *versus* multiple foci of microinvasion.

## Materials and Methods

We retrospectively reviewed the medical records of patients diagnosed with DCISM who underwent axillary staging at our institute from January 2006 to December 2015. The inclusion criteria were as follows: (1) female patients diagnosed with DCISM on the final pathology; (2) underwent axillary staging. Patients were excluded if they had: (1) neo-adjuvant chemotherapy prior to surgery or (2) history of breast cancer. Data on age, BMI, clinical features, type of surgery and type of axillary procedure, and any adjuvant radiation therapy, hormonal therapy or chemotherapy were collected. Pathologic variables collected included histologic tumor type, histologic and nuclear grade, presence of lymphovascular invasion (LVI), and receptor status [estrogen receptor (ER) and human epidermal growth factor receptor 2 (HER2)] in microinvasive sites. Lesion size on pathology was defined as total extent of breast lesion with both DCIS lesion and microinvasive foci. The study was approved by the Ethics Committee of Jiaxing University.

Patients in this study received surgical excision in our institution, including total mastectomy and breast conserving surgery (BCS). Microinvasion was defined as invasive portion no more than 1 mm. Pathological results were reviewed for statements regarding number of invasive foci and categorized as having one focus or multiple foci (≥2 foci). The cutoff was selected according to the language used in the pathology reports. Immunostaining for ER was performed, and cases with more than 1% were considered as positive staining. HER2 positivity was defined as cases where immunohistochemistry staining was 3+ or 2+ with fluorescence *in situ* hybridization positivity. Sentinel lymph node biopsy (SLNB) positivity was defined as the presence of micrometastasis (>200 cells or >0.2 mm, but <2.0 mm) or macrometastasis (>2.0 mm) identified on hematoxylin and eosin (H&E) staining. SLNs were identified *via* methylthionine chloride injection and were assessed *via* serial sections with 100 μm of spacing between sections.

Date and status at last follow-up were collected. Recurrence events were recorded as local-regional (ipsilateral breast and chest wall, ipsilateral axillary or supraclavicular lymph nodes) and distant. Time to recurrence and time to death were measured from date of surgery and censored at the date of last follow-up for event free patients. Time to recurrence was censored at time of death. Patients with less than 6 months follow-up after surgery were not included in the survival analysis. Loss to follow-up was defined as patients with less than 3-year follow-up after surgery.

The clinicopathological characteristics were compared between patients with one focus and multiple foci of microinvasion and between patients who received and did not receive chemotherapy using chi-square test for categorical variables. Time-to-event outcomes were estimated using Kaplan–Meier methods and were compared across groups using the log-rank test. Cox regression model was used for multivariate analysis in survival. All statistical analysis was performed using SPSS statistical software version 18.0 (IBM, Chicago, IL, USA), and P values less than 0.05 were considered significant.

## Results

A total of 359 patients with DCISM were identified, of which 233 (64.90%) had one focus of microinvasion and 126 (35.10%) had multiple foci. The median age of this cohort was 49 (range 26–83). The baseline characteristics of the cohort were shown in **Table 1**. Patients with multiple foci tended to have larger tumor size (*P* < 0.001), higher nuclear grade (*P* = 0.001), and higher rate of lymphatic vascular invasion (*P* = 0.034). Also, the proportion of positive HER2 (*P* = 0.027) and Ki67 level (*P* = 0.004) increased in patients with multiple foci. While in multivariate analysis, only tumor size showed significant difference (*P* = 0.009).

**Table 1:** Baseline characteristics, univariate and multivariate analysis of clinicopathological factors in DCISM patients with one focus and multiple foci.

Variables	Total N = 359	%	One focus N = 233	Multiple foci N = 126	Univariate *P*-value	Multivariate OR (95%CI), P-value
Age					0.874	
≤50	210	58.50	137	73		
>50	149	41.50	96	53		
BMI					0.792	
≤24	251	69.92	164	87		
>24	108	30.08	69	39		
Lump on PE					0.154	
No	90	25.07	64	26		
Yes	269	74.93	169	100		
Calcification					0.324	
No	129	35.93	88	41		
Yes	230	64.07	145	85		
Discharge					0.694	
No	307	85.52	198	109		
Yes	52	14.48	35	17		
Surgery type					0.089	
BCS	26	7.24	22	4		
Mastectomy	294	81.90	187	107		
Mastectomy +BR	39	10.86	24	15		
Axillary evaluation					0.244	
SLNB	242	67.41	162	80		
ALND	117	32.59	71	46		
Size on pathology					<0.001	
≤2 cm	114	31.75	86	28		Ref
>2 cm	221	61.56	125	96		2.007 (1.190–3.385), 0.009
NA	24	7.69	22	2		0.343 (0.073–1.616), 0.176
Grade					0.001	
Low	23	6.41	19	4		Ref
Medium	105	29.25	73	32		1.846 (0.532–6.402), 0.334
High	194	54.04	110	84		2.638 (0.755–9.215), 0.129
NA	37	10.30	31	6		1.151 (0.263–5.038), 0.852
LVI					0.034	
No	354	98.61	232	122		Ref
Yes	5	1.39	1	4		7.386 (0.740–73.686), 0.088
ER					0.231	
Negative	147	40.95	88	59		
Positive	207	57.66	142	65		
NA	5	1.39	3	2		
HER2					0.027	
Negative	100	27.86	73	27		Ref
Positive	205	57.10	121	84		1.151 (0.623–2.126), 0.654
NA	54	15.04	39	15		1.131 (0.506–2.526), 0.764
Ki67					0.004	
≤14%	82	22.84	63	19		Ref
>14%	192	53.48	110	82		1.717 (0.900–3.276), 0.101
NA	85	23.68	60	25		1.354 (0.639–2.870), 0.429

DCISM, ductal carcinoma in situ with microinvasion; OR, odd ratio; CI, confidence interval; BMI, body mass index; PE, physical examination; BCS, breast conserving surgery; BR, breast reconstruction; SLNB, sentinel lymph node biopsy; ALND, axillary lymph nodes dissection; NA, not available; LVI, lymphovascular invasion; ER, estrogen receptor; HER2, human epidermal growth factor receptor 2.

Overall, 242 (67.41%) and 117 (32.59%) patients underwent sentinel lymph nodes biopsy (SLNB) and axillary lymph nodes dissection (ALND), respectively. The medium number of SLNs was three [interquartile range (IQR) 2–4]. Isolated tumor cells (ITCs) were found in four (1.11%) patients and axillary metastasis rate was 2.51%. Neither axillary evaluation methods (*P* = 0.244) nor axillary metastasis rate (2.07 *vs* 3.42%, *P* = 0.559) was significantly different between patients with one focus and multiple foci (**Table 2**).

**Table 2:** Number of microinvasive foci and rate of axillary metastasis with *P* values for comparison of rates between groups.

No. of microinvasive foci	No. of patients	No. of patients with ITCs	No. of patients with axillary metastasis
One	242	2 (0.83%)	5 (2.07%)
Multiple	117	2 (1.71%) *P* = 0.578	4 (3.42%) *P* = 0.559
Total	359	4 (1.11%)	9 (2.51%)

ITC, isolated tumor cells.

For patients who received BCS, 53.85% (14/26) received adjuvant radiation therapy. There were 17 patients who received adjuvant radiation therapy; all of them had one focus of microinvasion. There were differences in receipt of adjuvant systemic therapy in the two groups. Hormonal therapy was used more frequently in the one -focus group (49.36 *vs* 35.71%; *P* = 0.051), while chemotherapy was used more frequently in the multiple-foci group (56.35 *vs* 40.77%; *P* = 0.028), which can partly be explained by the different clinicopathological factors in these two groups. Also, we found that both the use of hormonal therapy and chemotherapy was more common among patients with positive axillary. Hormonal therapy was given to 55.56% of axillary-positive patients compared to 44.29% of axillary-negative patients (*P* = 0.111), which did not reach significant differences. Moreover, 66.67 and 45.71% of axillary-positive and axillary-negative patients received chemotherapy, respectively (*P* = 0.003).

In the subset of patients with negative axillary, multiple foci of microinvasion were also associated with adjuvant systemic therapy, suggesting that this pathologic factor may be used in decision making for adjuvant systemic therapy. Compared with patients with one focus of microinvasion, patients with multiple foci were more likely to receive chemotherapy: 55.74% (68/122) *vs* 40.35% (92/228), respectively (*P* = 0.029). However, they were less likely to receive hormonal therapy: 33.61% (41/122) *vs* 50.00% (114/228), respectively (*P* = 0.019). Furthermore, we divided patients with negative axillary into two groups by the usage of chemotherapy to further investigate whether the number of microinvasive foci was independently associated with chemotherapy (**Table 3**). In the univariate analysis, we found that most of the pathological factors were associated with chemotherapy. However, only tumor size, ER, and HER2 showed significant differences in multivariate analysis. Patients with larger tumor size (*P* = 0.008), negative ER (*P* = 0.023) and positive HER2 (*P <* 0.001) were more likely to receive chemotherapy. Thus, the number of microinvasive foci was not significantly associated with chemotherapy in patients with negative axillary.

**Table 3:** Univariate and multivariate analysis of impact factors on chemotherapy in DCISM patients with negative axillary.

Variables	Chemo− N = 190	%	Chemo+ N=160	%	Univariate *P*-value	Multivariate OR (95%CI), P-value
Size on pathology					<0.001	
≤2 cm	75	39.47	35	21.87		Ref
>2 cm	99	52.11	117	73.13		2.025 (1.198–3.423), 0.008
NA	16	8.42	8	5.00		1.148 (0.408–3.232), 0.793
Grade					0.003	
Low	19	10.00	3	1.87		Ref
Medium	58	30.53	45	28.13		2.820 (0.743–10.703), 0.128
High	90	47.37	99	61.88		2.008 (0.516–7.808), 0.315
NA	23	12.10	13	8.12		2.993 (0.695–12.885), 0.141
LVI					<0.001	
No	188	98.95	158	98.75		Ref
Yes	2	1.05	2	1.25		2.108 (0.224–19.848), 0.515
Number of foci					0.002	
One focus	136	71.58	92	57.50		Ref
Multiple foci	54	28.42	68	42.50		1.427 (0.870–2.340), 0.159
ER					<0.001	
Negative	57	30.00	88	55.00		Ref
Positive	130	68.42	71	44.38		0.554 (0.333–0.923), 0.023
NA	3	1.58	1	0.62		0.687 (0.062–7.632), 0.760
HER2					<0.001	
Negative	71	37.37	25	15.63		Ref
Positive	82	43.16	120	75.00		3.167 (1.697–5.913), <0.001
NA	37	19.47	15	9.37		1.146 (0.521–2.522), 0.735
Ki67					0.182	
≤14%	50	26.32	31	19.37		
>14%	94	49.47	94	58.75		
NA	46	24.21	35	21.88		

DCISM, ductal carcinoma in situ with microinvasion; OR, odd ratio; CI, confidence interval; NA, not available; LVI, lymphovascular invasion; ER, estrogen receptor; HER2, human epidermal growth factor receptor 2.

**Figure 1:**
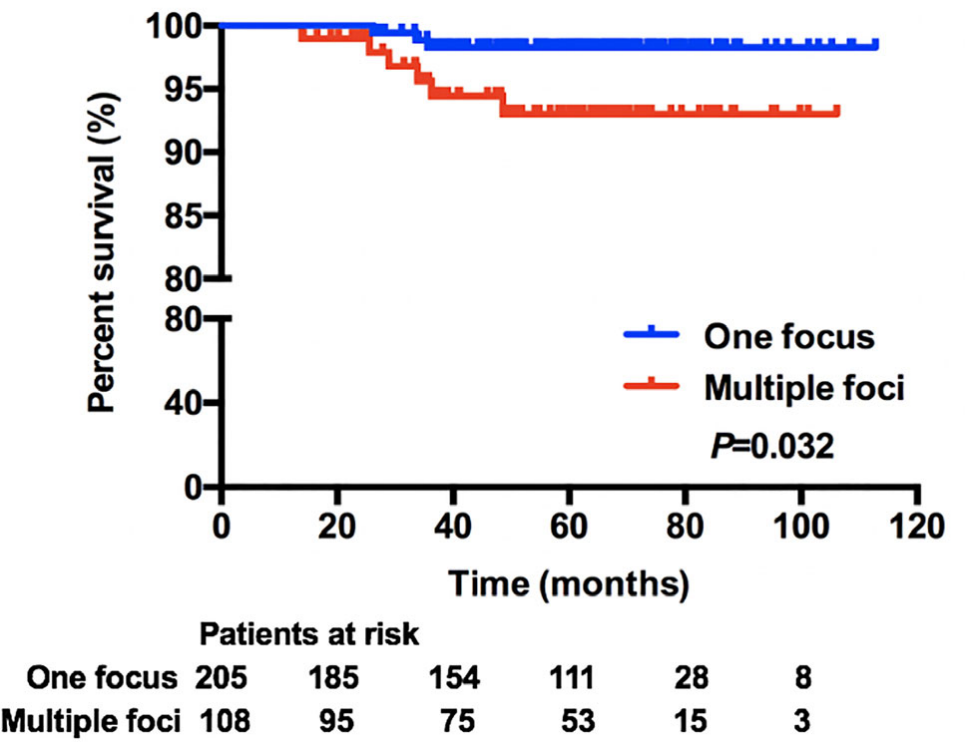
Comparison of DFS between one-focus and multiple-foci patients.

In this cohort, 313 (87.19%) patients were included in the survival analysis. The median follow-up was 61.27 months, which was 5.11 years for the total population and was similar for one-focus and multiple-foci groups. Overall survival rate was 99.36% for the total population. There were nine (2.88%) recurrences: four local and regional; four distant; and one concurrent local and distant (**Supplementary Table 1**). Kaplan–Meier analysis showed that patients with multiple microinvasive foci had worse DFS rate compared with one-focus patients (98.29 *vs* 93.01%, *P* = 0.032) (**Figure 1**). While, multivariate analysis showed that the only independent predictor for worse DFS was axillary metastasis status (**Table 4**). Compared with patients with no axillary metastasis, patients with positive axillary were 26.70-fold more likely to have worse DFS (*P* = 0.016).

**Table 4:** Multivariate analysis *via* Cox regression model for independent predictors on DFS.

Variables	Values	*P* value, OR (95% CI)
Age	≤50 *vs* >50	0.419, 2.048 (0.360–11.652)
Size on pathology	>2 cm *vs* ≤2 cm	0.451, 2.545 (0.225–28.817)
Grade	High *vs* low and medium	0.799, 0.799 (0.143–4.463)
LVI	Positive *vs* negative	0.451, 3.836 (0.117–126.135)
Number of foci	Multiple *vs* one	0.376, 2.173 (0.390–12.106)
ER	Positive *vs* negative	0.088, 0.108 (0.008–1.397)
HER2	Positive *vs* negative	0.382, 2.816 (0.276–28.736)
LN status	Positive *vs* negative	0.016, 26.700 (1.823–391.150)
Adjuvant chemotherapy	No *vs* yes	0.291, 0.228 (0.015–3.549)
Adjuvant hormonal therapy	No *vs* yes	0.297, 0.335 (0.043–2.612)

DFS, disease-free survival; OR, odd ratio; CI, confidence interval; LVI, lymphovascular invasion; ER, estrogen receptor; HER2, human epidermal growth factor receptor 2; LN, lymph nodes.

## Discussion

DCISM is rare, and there are controversial results reported on the outcomes of this subtype compared with pure DCIS and invasive breast cancer. Generally, the biological behavior and survival outcomes of DCISM were intermediate between those diagnosed with DCIS and invasive breast cancer. However, previous studies have shown conflicting results on both axillary metastasis and survival outcome of DCISM patients. In this current study, we focused on the association between extent of microinvasion foci in DCIS with axillary status and survival outcome.

Previous studies showed that the biological behavior of DCISM was worse than pure DCIS, such as larger tumors, higher tumor grade, human epidermal growth factor receptor 2 positivity, and a high Ki-67 index (14, 17, 18). In our study, we also found that DCIS patients with multiple foci of microinvasion had more aggressive biology than those with one focus of microinvasion. Patients with multiple foci tended to have larger tumor size, higher nuclear grade, and higher rate of lymphatic vascular invasion, which were all potential impact factors of worse prognosis. Also, there were more HER2 positivity and higher Ki67 level in patients with multiple foci. In multivariate analysis, we found that only tumor size showed a significant difference, indicating that DCIS patients with larger tumor (>2 cm) were more likely to have multiple invasive foci.

Currently, the extent of microinvasion in DCIS is not a factor included in the staging of breast cancer. Although the size of the tumor has clearly been associated with axillary status, and overall prognosis in patients diagnosed with invasive breast cancer, multiple foci of microinvasion in DCIS have not been consistently linked to outcomes above (13, 19). It seems possible that an increased disease burden with multiple foci of microinvasion can lead to worse outcomes, yet due to the rarity of this specific pathological pattern, the impact of the extent of microinvasion in DCIS on axillary metastasis and patient survival outcome has not been clearly described. Cindy B. Matsen et al. reported that extent of microinvasion in DCIS was not associated with axillary metastasis, showing that there was no higher risk of nodal involvement with multiple foci of microinvasion as compared to one focus (20). While, in other research, rates of axillary involvement differed in DCIS patients with different numbers of microinvasive foci, showing that 2.1% axillary metastasis in patients with one focus and 15.6% for multiple foci (*P* = 0.037) (21). There were few data on the long-term outcome of this specific pathological subtype, and results were inconsistent. Some indicated that different extent of microinvasive foci in DCIS had similar survival outcome, while others did not (21–23). A study from Canada showed that multiple foci of microinvasion were associated with higher risk of invasive local recurrence in DCIS patients received BCS (23). Another study also showed that multifocality in DCIS was associated with a worse survival in chemotherapy and trastuzumab-naive patients, while in multivariate regression analysis, the difference was statistically insignificant (21). In our study, the incidence of pathologically positive axillary metastasis in DCISM patients was 2.51%, which was comparable with previous studies (21, 24). We found that the axillary metastasis rate was similar in patients with one focus and multiple foci (2.07 *vs* 3.42%, *P* = 0.559). However, the extent of microinvasion was associated with DFS in DCIS patients. Compared with one-focus patients, patients with multiple foci had worse DFS rate (93.01 *vs* 98.29%, *P* = 0.032).

Notably, in our study, the mastectomy rate and ALND rate were both high. Over 80% of DCISM patients received mastectomy, and over 30% received ALND. This may be related to the characteristic of DCIS on preoperative imaging and potential underestimate of invasive breast cancer in patients diagnosed with DCIS by core needle biopsy. As we have known, the extent of DCIS lesions on preoperative imaging can be large and multicentric. Also, microinvasive foci were more likely to be found in larger background of DCIS than smaller lesion. Thus, these DCISM patients were more likely to receive mastectomy than BCS. Furthermore, the potential upstaging of DCISM to invasive breast cancer was an important issue for surgical options, especially axillary evaluation. Previous studies showed that about 14–32% of patients diagnosed with DCIS on core needle biopsy could be upstaged to invasive diseases after surgical excision (25–27). Also, the volume of breasts in eastern patients were smaller than western patients, which led to less BCS. And the increasing application of breast reconstruction was another important issue for surgical choices preferring mastectomy.

In this current study, we found that the usage of adjuvant therapy was different in patients with one focus or multiple foci of microinvasion, mostly due to the different clinicopathological factors in these two groups. All patients received adjuvant radiation therapy had one focus of microinvasion. Patients with one focus of microinvasion were more likely to had smaller tumor, which led to more possibility of BCS. Thus, adjuvant radiation therapy was more common among patients with one focus of microinvasion. Also, we found that both hormonal therapy and chemotherapy were more common among patients with positive axillary, which was reasonable because nodal metastasis was one of the powerful predictors of recurrence and survival in breast cancer. In patients with negative axillary, the extent of microinvasion in DCIS was not associated with adjuvant chemotherapy, while tumor size, ER, and HER2 did appear to influence this decision. Thus, multiple microinvasions alone was not a significant impact factor for decision on adjuvant chemotherapy.

For small breast tumors, especially DCIS or DCISM, whether they receive adjuvant therapy is always a dilemma. Some previous studies showed that patients with small tumors receiving systemic therapy were significantly younger and had lymph node metastasis, higher tumor grade, negative ER, and positive HER2 status (28–30). While other studies showed that no benefit was observed for adjuvant chemotherapy in very small tumors, such as T1mi, T1a, and T1b HER2-positive or triple-negative breast cancers with no axillary metastasis (31, 32). Also, studies showed that either aromatase inhibitors or tamoxifen could be an effective adjuvant treatment options in order to lower the risk of recurrent DCIS (33, 34). For DCIS or DCISM patients who received BCS, radiation therapy remains to be the standard of care (35, 36). Up to now, whether it is necessary to receive chemotherapy and target therapy in DCISM patients has not been addressed clearly. According to our findings, patients with larger tumor (>2cm), negative ER, and positive HER2 were more likely to receive chemotherapy. Also, previous studies have proved that tumor size, ER negativity, and HER2 overexpression promoted factors on invasion and metastasis in breast cancer (37, 38). Thus, we suggest that, in high-risk DCISM patients, adjuvant systemic therapy can be properly applied.

Interestingly, we found that almost all the recurrence patients in the cohort had overexpressed HER2, which was well-known by accelerating cell proliferation and enhancing malignant behavior (39, 40). For HER2 positive patients with adjuvant chemotherapy (n = 122), only 14 patients also administered Trastuzumab. With limited cases, we still found that patients who received Trastuzumab had no recurrence in a median follow-up of 24.80 months, while patients without Trastuzumab had 5.66% recurrence rate in a median follow-up of 24.20 months. In addition, compared with patients without Trastuzumab, patients received Trastuzumab had larger tumor size on average (3.9 *vs* 3.6 cm) and higher rate of multiple foci of microinvasion (64.3 *vs* 43.5%). Thus, HER2-positive patients with multiple foci of microinvasion could be considered for optimization of target therapy. Previous studies reported that HER2 overexpression was an independent predictor of invasive disease and poor prognosis, indicating that HER2 expression might be associated with an important pathway through which DCIS lesions may progress toward invasive lesions (18, 21, 41). Thus, HER2 overexpression in DCIS patients might be a predictor of the presence of invasive foci and long-term recurrence after surgery. For HER2 positive DCISM patients with other high risks of poor outcome, both adjuvant chemotherapy and target therapy could be considered.

There were several limitations to the present study. First, it was a retrospective study, which led to lower level of evidence. However, this study had a relatively large dataset with uniform inclusion and exclusion criteria. Second, some information was missing, which could lead to bias in the analysis. Furthermore, the pathological features were mixed. Grading criteria were for *in situ* lesions, while ER and HER2 status was in microinvasive sites because the invasive component was too small to adequately grade. Further assessment is needed to accurately select low-risk DCISM patients who can safely avoid axillary staging and adjuvant systemic therapy.

## Conclusion

DCIS with microinvasion is a rare subgroup in breast cancer patients and presents a therapeutic conundrum. Even though the numbers of microinvasion were different and patients with multiple foci of microinvasion tended to have larger tumor size, there was no higher risk of axillary involvement compared with patients with one focus of microinvasion. While, patients with multiple microinvasive foci had worse DFS rate. Thus, DCISM patients with multiple foci of microinvasion may be the criterion for more aggressive local–regional treatment, especially in HER2 positive patients. Optimization of adjuvant therapy in DCISM patients is required.

## Data Availability Statement

The original contributions presented in the study are included in the article/**Supplementary Material**. Further inquiries can be directed to the corresponding author.

## Ethics Statement

The studies involving human participants were reviewed and approved by First Hospital of Jiaxing. The patients/participants provided their written informed consent to participate in this study.

## Author Contributions

JS wrote this article. RG did the statistics. HP, XL, ZG, C, LX, and DX collected data. WW and CC gave administration support. All authors contributed to the article and approved the submitted version.

## Funding

This work was supported by grants from National Natural Science Foundation of China (Grant No. 81902674) and Innovation Subject of Jiaxing (Grant No. 2019-cx-04).

## Conflict of Interest

The authors declare that the research was conducted in the absence of any commercial or financial relationships that could be construed as a potential conflict of interest.
