# Supplementary material for: Multiple Microinvasion Foci in Ductal Carcinoma In Situ Is Associated With an Increased Risk of Recurrence and Worse Survival Outcome
Source: Front Oncol. 2020 Dec 3;10:607502. doi: 10.3389/fonc.2020.607502 (PMC7744719; doi:10.3389/fonc.2020.607502)
Supplement: Supplementary file 1 [file DataSheet_1.docx]

Supplementary Table 1 Clinicopathological features of 9 recurrence patients in this cohort.

| Patient number | Age | Surgery type | Axillary evaluation | Size (cm) | Grade | Focus/foci | Axillary status | LVI | ER | HER2 | Ki67 | Hormonal therapy | Chemotherapy | Target therapy | recurrence |
| --- | --- | --- | --- | --- | --- | --- | --- | --- | --- | --- | --- | --- | --- | --- | --- |
| 1 | 54 | M+BR | SLNB | 6.0 | NA | Multiple | Negative | N | Positive | Positive | ≤14% | Y | Y | N | Distant |
| 2 | 54 | M | ALND | 5.0 | High | Multiple | Positive | Y | Positive | Positive | >14% | Y | Y | N | Distant |
| 3 | 48 | M | SLNB | 1.2 | Medium | One | Negative | N | Negative | Positive | NA | Y | Y | N | Distant |
| 4 | 41 | BCS | ALND | 2.8 | Medium | One | Negative | N | Negative | Positive | ≤14% | N | Y | N | Local and regional |
| 5 | 36 | M+BR | ALND | 3.0 | High | Multiple | Negative | N | Negative | Positive | >14% | N | Y | N | Local and regional |
| 6 | 33 | M+BR | ALND | 2.5 | High | One | Negative | N | Negative | Positive | NA | N | Y | N | Distant |
| 7 | 65 | M | SLNB | 2.5 | High | Multiple | Negative | N | Negative | Positive | >14% | N | N | N | Local and regional |
| 8 | 35 | M+BR | ALND | 3.0 | High | Multiple | Positive | N | Positive | NA | >14% | Y | Y | N | Local and regional |
| 9 | 56 | M | ALND | 2.5 | High | Multiple | Negative | N | Negative | Positive | >14% | N | Y | Y | Local and distant |

Abbreviations: M, mastectomy; BR, breast reconstruction; BCS, breast conserving surgery; SLNB, sentinel lymph node biopsy; ALND, axillary lymph nodes dissection; NA, not available; Y, yes; N, no.
